# The effect and the mechanism of allo-bone marrow mesenchymal stem cells in experimental autoimmune thyroiditis

**DOI:** 10.1186/1687-9856-2015-S1-O29

**Published:** 2015-04-28

**Authors:** Rongxiu Zheng, Yanan Chu, Lihui Wang, Yongqing Ni, Yi Zhang

**Affiliations:** 1 Department of Pediatrics, Tianjin Medical University General Hospital, Tianjin, China; 2 Department of Cell Biology, Institute of Basic Medical Sciences, Beijing, China

## Aims

To investigate the effect and the mechanism of allo-bone marrow mesenchymal stem cells (BM-MSCs) transplantation in the Experimental Autoimmune Thyroiditis (EAT) mouse model.

## Methods

C57BL/6 mice were treated with porcine thyroglobulin and Freund's adjuvant both in the simple model group and the BM-MSCs treated group. Mice in the BM-MSCs treated group were injected with homology BM-MSCs(3×10^5^/mouse) at the beginning of model establishment. A normal control group was set as well. All mice were killed at the 28th day after primary immunity. The histopathological analysis of thyroid tissues were observed. The levels of autoantibodies, thyroid hormone, IFN-r, IL-10 were detected.

## Results

(1)) A mouse model of EAT was established successfully. (2) The thyroid in the simple model group and the BM-MSCs treated group showed inflammatory response and inflammatory cell infiltration, but the response in the BM-SCs treated group was weaker than in the simple model group. [3] Compared with the normal control group, increased autoantibodies were detected with P<0.05, an increased IFN-γ level and a decrease of IL-10 in the simple model group; Compared with the simple model group, decreased autoantibodies were detected with P<0.05 in the BM-MSCs treated group; Decreased expression of IFN-γ and increased expression of IL-10 was detected in this group with P<0.05.

## Conclusion

BM-MSCs could partly restore the immunological homeostatic state through modulating the immunological dissonance of Th1/Th2, and alleviate inflammation of the thyroid. It may provide a new approach to therapy of autoimmune thyroiditis.

